# Comparison of Techniques for Obtaining Centric Relation Based on the Reproducibility of the Condylar Positions in Centric Relation—A Systematic Review

**DOI:** 10.1055/s-0041-1735903

**Published:** 2021-12-17

**Authors:** Clóvis Lamartine de Moraes Melo Neto, Daniela Micheline dos Santos, André Pinheiro de Magalhães Bertoz, André Luiz de Melo Moreno, Marcelo Coelho Goiato

**Affiliations:** 1 Department of Dental Materials and Prosthodontics, School of Dentistry, São Paulo State University, Araçatuba, São Paulo, Brazil; 2 Oral Oncology Center, School of Dentistry, São Paulo State University, Araçatuba, São Paulo, Brazil; 3 Department of Pediatric and Social Dentistry, School of Dentistry, São Paulo State University, Araçatuba, São Paulo, Brazil

**Keywords:** dental centric relation, reproducibility of findings, jaw relation record, chin point guidance, bimanual manipulation, gothic arch, swallowing

## Abstract

The objective of this systematic review was to compare centric relation (CR) techniques that belong to the same method of obtaining CR (guided, graphical, or physiological method), to verify which CR technique within each method of obtaining CR generates the greatest reproducibility of the condylar positions (or mandibular position) in CR. The PubMed, Cochrane Library, SciELO, Scopus, and Web of Science databases were searched for articles published up to May 5, 2021. The search terms were combinations of “dental centric relation” (MeSH), with each of the following terms (individually): “reproducibility of findings” (MeSH); “jaw relation record” (MeSH); “chin point”; “gothic arch”; “bimanual manipulation”; “swallowing” (MeSH); and “jig.” Inclusion criteria: clinical studies in English; individuals without temporomandibular dysfunction and with complete or almost complete dentition or complete edentulous; and comparison between CR techniques belonging to the same method of obtaining CR based on the reproducibility of condylar positions in CR. For each method of obtaining the CR, the following CR techniques were considered: guided method (chin point guidance and bimanual manipulation); graphic method (intraoral and extraoral gothic arch tracing); and physiologic method (swallowing and tongue retrusion along the palate). A total of 1692 articles were screened. After the inclusion and exclusion criteria were applied, six articles were included in this review. None of the included studies evaluated edentulous individuals. All included articles compared CR techniques of the guided method. Three articles concluded that the bimanual technique showed greater reproducibility of the condylar positions in CR than the chin point guidance technique, two articles showed equivalence between these techniques, and 1 article concluded that the chin point guidance technique showed greater reproducibility of the condylar positions in CR than the bimanual technique. Thus, in this systematic review, the bimanual technique was often superior (generated greater reproducibility of the CR) or at least equivalent to the chin point guidance technique. Therefore, for individuals with complete dentition and without temporomandibular disorders, the bimanual technique is more recommended.

## Introduction

The definition of centric relation (CR) has been redefined several times over the years. 1 2 3 The most recent edition of the Glossary of Prosthodontic Terms states that CR is “a maxillomandibular relationship, independent of tooth contact, in which the condyles articulate in the anterior-superior position against the posterior slopes of the articular eminences; in this position, the mandible is restricted to a purely rotary movement; from this unstrained, physiologic, maxillomandibular relationship, the patient can make vertical, lateral or protrusive movements; it is a clinically useful, repeatable reference position.” 1 2

CR is inherently individual to each person. 2 Thus, regardless of the definition of CR, 1 3 the dentist must gently guide the patient's mandible in the direction posterior to the maximum intercuspation, until the patient's anatomical components (e.g., muscles, ligaments, condyles, and discs) and physiological limits establish his or her CR. It is worth mentioning that CR must not be forcibly established. 2

CR is important for both dentate and edentulous individuals, because it plays an important role in prosthetic rehabilitation, temporomandibular disorder (TMD) therapy, orthodontic and maxillofacial planning, occlusal rehabilitation, and maintenance of oral health. 2 The ultimate goal of the CR record is to achieve harmonious relationships between teeth, joints, and muscles. 2 Therefore, a CR technique that generates the greatest reproducibility of condylar positions in CR plays an extremely important role in clinical practice. 2

The most commonly used CR classical techniques are bilateral manipulation and chin point guidance, which are considered guided methods (i.e., they belong to the guided method of obtaining CR) 2 4 ; intraoral and extraoral gothic arch tracing, which are considered graphic methods (i.e., they belong to the graphic method of obtaining CR) 2 4 ; and the swallowing technique and tongue retrusion along the palate, which are considered physiologic methods (i.e., they belong to the physiologic method of obtaining CR). 2 4

In a recent systematic review, de Moraes Melo Neto et al compared classical CR techniques of different methods of obtaining CR to verify which CR technique generated the greatest reproducibility of the condylar positions in CR. 2 Thus, the present study aims to assess another situation; that is, the objective of this systematic review is to compare CR techniques that belong to the same method of obtaining CR (guided, graphical, or physiological method), to verify which CR technique within each method of obtaining CR generates the greatest reproducibility of the condylar positions (or mandibular position) in CR.

## Methods

This study followed the Preferred Reporting Items for Systematic Reviews and Meta-Analyses (PRISMA) criteria proposed for a systematic review. 5 This systematic review was registered on PROSPERO (international prospective register of systematic reviews, CRD42018097285) at the beginning of the study. Then, the population (P), intervention (I), comparison (C), and outcome (O) were determined to form a research question:

➢ **P** —individuals submitted to techniques for obtaining CR.
➢ **I** —techniques for obtaining CR.
➢ **C** —Comparison between CR techniques belonging to the same method of obtaining CR, based on the reproducibility of the condylar positions (or mandibular position) in CR.
➢ **O** —The expected result is to know which CR technique, within each method of obtaining the CR, can generate the greatest reproducibility of the CR.

### Search Strategy and Selection of Studies

Two calibrated independent reviewers 2 (C.L.d.M.M.N. and M.C.G.) conducted a search in the PubMed, Cochrane Library, SciELO, Scopus, and Web of Science databases for relevant articles published up to May 5, 2021. The search terms were combinations of “dental centric relation” (MeSH) with each of the following terms (individually): “reproducibility of findings” (MeSH); “jaw relation record” (MeSH); “chin point”; “gothic arch”; “bimanual manipulation”; “swallowing” (MeSH); and “jig.” 2 After duplicate articles were excluded, the titles and abstracts of all potentially eligible studies were screened, according to the inclusion and exclusion criteria, and studies that clearly did not meet the criteria were eliminated. 2 The full text of each remaining preselected article was then evaluated, according to the inclusion and exclusion criteria, and those articles that met these criteria were included in this systematic review. 2

### Inclusion Criteria

□ Clinical studies in English evaluating individuals without TMD. 2
□ Individuals with complete or nearly complete dentition (angle class I, II, or III) or complete edentulism. 2
□ Studies must evaluate at least five individuals. 2
□ Studies must compare at least two CR techniques belonging to the same method of obtaining CR, based on the reproducibility of CR.
□ For each method of obtaining CR, the following CR techniques were considered:
❖ Graphic method—Intraoral and extraoral gothic arch tracing were considered. 2
❖ Physiologic method—The swallowing technique and tongue retrusion along the palate were considered. 2 4
❖ Guided method—Bimanual manipulation and chin point guidance were considered. 2 4 As in the study by de Moraes Melo Neto et al, 2 the chin point guidance technique could be its traditional version 4 and its modified version (three finger chin point guidance technique). 4

### Exclusion Criteria

□ Duplicated studies. 2
□ Removable partial denture wearers. 2
□ Studies that did not inform the individual's dental condition. 2
□ Clinical studies with incomplete data. 2
□ Literature reviews. 2
□ Systematic reviews. 2
□ Case reports. 2

### Kappa Method

The agreement between the two reviewers based on the evaluation of titles and abstracts, and later on the evaluation of the full text of the articles, was evaluated using the Kappa statistic.

### Quality Assessment

The quality of the studies was assessed with the Jadad scale. 2 6 This scale evaluates if the study is randomized and double-blind, if these factors are well described, and if there is a description of dropouts in each study. 2 6 For each question, the answer can be “yes” (1 point) or “no” (0 points). 2 6 After the sum of the scores, the study is considered of low quality if the result is from 0 to 2, or high quality if the result is from 3 to 5. 2 6

### Data Analysis

The following data were collected from the studies: sex and age of participants; ^2^ sample size; ^2^ dental condition of the participants (dentate or edentulous); ^2^ techniques used for obtaining CR; ^2^ whether randomization of techniques was performed; ^2^ period of the day for obtaining CR; ^2^ the position of the individual during CR registration; ^2^ the number of repetitions of CR techniques; ^2^ the instrument or apparatus used to compare CR techniques; ^2^ and the number of operators. ^2^

## Results

**Fig. 1:**
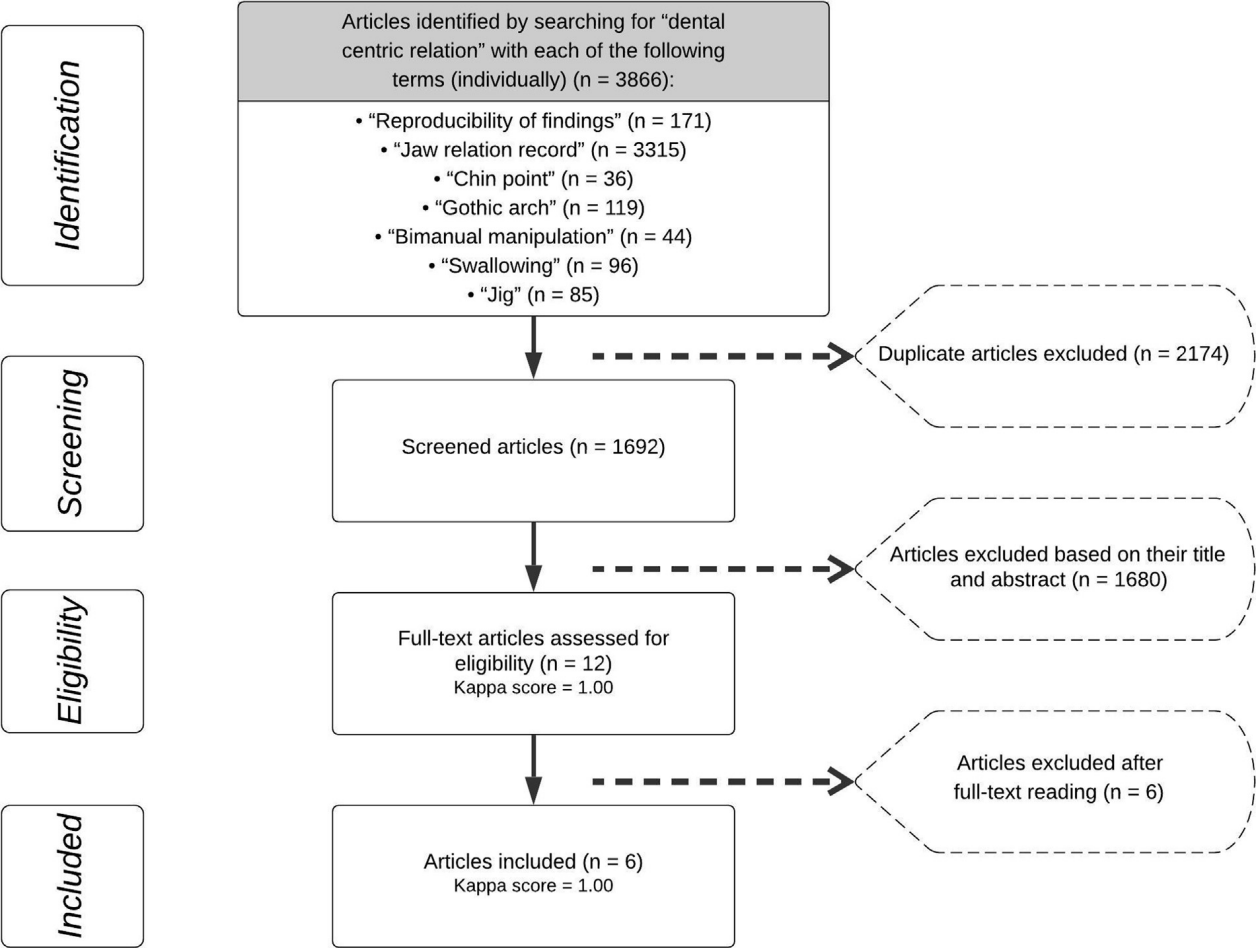
PRISMA flowchart showing the process of identification, screening, eligibility and inclusion of articles.

From an initial total of 1692 identified studies, 12 articles 7 8 9 10 11 12 13 14 15 16 17 18 were selected for full-text analysis (Kappa score = 1.00), resulting in the inclusion of six clinical studies 7 8 9 10 11 12 (Kappa score = 1.00) that met the inclusion and exclusion criteria ( Fig. 1 ). The quality analysis of the studies according to the Jadad scale showed that all articles were of low quality ( Table 1 ). The reasons for exclusion of the other articles that received a full-text review are indicated in Table 2 . 13 14 15 16 17 18 Tables 3 and 4 show the data collected from the six articles included in this systematic review. 7 8 9 10 11 12

**Table 1:** Scores reached by the articles following the criteria of the Jadad scale

Jadad scale	Kantor et al 7	Simon and Nicholls 8	Teo and Wise 9	Hobo and Iwata 10	Keshvad and Winstanley 11	Galeković et al 12
1.Was the study described as randomized?	0	1	1	0	0	0
2.Was the randomization described and appropriate?	0	1	1	0	0	0
3. Was the study described as double-blind?	NA	NA	NA	NA	NA	NA
4. Was the double-blind method appropriate?	NA	NA	NA	NA	NA	NA
5 Was there a description of withdrawals and dropouts?	0	0	0	0	0	0
Results	0	2	2	0	0	0
Quality of study	Low	Low	Low	Low	Low	Low

Abbreviation: NA, not applicable.

**Table 2:** Reason for exclusion after reading the articles in full

Reason	References
Repeated article	Kantor et al 13
Studies that did not compare CR techniques based on the reproducibility of condylar positions in CR	Carwell and Mcfall, 14 McWilliam, 15 Hellsing et al 16 and Watanabe 17
Lack of information about the technique	McKee 18

Abbreviation: CR, centric relation.

**Table 3:** Part 1 of data collection of selected article

Author/year	Total of individuals	Women( *n* )	Men( *n* )	Age	Dental condition	Guided method	Graphic method	Physiological method
Kantor et al 7	15	3	12	21–45	Complete dentition a	Chin point guidance with and without JIG/Bimanual	NE	NE
Simon and Nicholls 8	5	5	0	Third decade of life	Complete dentition a	Chin point guidance (JIG)/Bimanual (JIG)	NE	NE
Teo and Wise 9	7	NR	NR	17–29	Complete dentition a	Chin point guidance (JIG)/Bimanual (JIG)	NE	NE
Hobo and Iwata 10	10	NR	NR	21–32	Complete dentition a	Chin point guidance (JIG)/Bimanual (JIG)	NE	NE
Keshvad and Winstanley 11	14	7	7	26.61±4.2	Complete dentition a	Chin point guidance (JIG)/Bimanual (JIG)	NE	NE
Galeković et al 12	32	16	16	20–33	Complete dentition b	Chin point guidance (JIG)/Bimanual (JIG)	NE	NE

Abbreviations: CR, cenric relation; NE, not evaluated; NR, not reported.

JIG—This means that the jig was used to deprogram proprioceptive memory.

Galeković et al reported that the jig used by them was a cotton pellet. The other studies used Lucia's jig.

Chin point guidance with ramus support (Simon and Nicholls) and chin point guidance associated with applied muscle force by the subject (Teo and Wise) were not considered in this systematic review.

a Angle classification not provided.

b Angle class I.

**Table 4:** Part 2 of data collection of selected articles

Authors	Randomization of techniques	Evaluation period	Patient position	Number of records per technique	Evaluation apparatus	Number of operators
Kantor et al 7	NR	NR	NR	6	Articulator and mechanical microscope	NR
Simon and Nicholls 8	Yes	NR	NR	5	Custom aluminum plate with three measuring points	NR
Teo and Wise 9	Yes	NR	Supine	3	Whip-Mix articulator, instrument based on the Buhnergraph, and microscope	NR
Hobo and Iwata 10	NR	NR	NR	3	Experimental electronic mandibular recording microcomputer	1
Keshvad and Winstanley 11	NR	at approximately the same time of day	Upright	4 (initially, after 1 hour, after 1 day, and after 1 week)	Articulator (Denar D4A), custom-made mandibular position indicator, and stereomicroscope modified (Olympus OM-2)	1
Galeković et al 12	NR	at approximately the same time of day	NR	4 (initially, the next day, after 1 week, and after 1 month)	Mandibular position indicator (SAM Prazisionstechnik GmbH)	NR

Abbreviation: NR, not reported.

None of the six included studies evaluated edentulous individuals. Therefore, the six articles included evaluated individuals with complete dentition ( Table 3 ). 7 8 9 10 11 12 All comparisons were only between the bimanual and chin point guidance techniques. 7 8 9 10 11 12 Kantor et al, Hobo and Iwata, and Keshvad and Winstanley found that the bimanual technique generated greater reproducibility of the condylar positions in CR when compared with the chin point guidance technique. 7 10 11 Simon and Nicholls and Galeković et al found that there was no difference between these techniques, based on the reproducibility of condylar positions in CR. 8 12 Only Teo and Wise found that the chin point guidance technique generated greater reproducibility of the condylar positions in CR compared with the bimanual technique. 9

## Discussion

All studies included in this systematic review were considered of poor quality, according to the Jadad scale. 6 Despite this, studies comparing CR techniques cannot be double-blind, since the operator must know which CR technique will be performed on the patient. 6

Only Simon and Nicholls and Teo and Wise performed the CR techniques randomly. 8 9 Randomization is considered the gold standard for determining the effectiveness of medical/dental interventions in clinical research, and it should have been used in all studies included in this systematic review. 2

Kantor et al, Hobo and Iwata, and Keshvad and Winstanley observed that the bimanual technique generated greater reproducibility of the condylar positions in CR than the chin point guidance technique. 7 10 11 Therefore, half of the articles included in this systematic review showed a superiority of the bimanual technique. 7 10 11 Theoretically, this result can have occurred because, when performing the bimanual technique, the dentist can stabilize and guide the patient's mandible more efficiently with her or his hands compared with the chin point guidance technique, in which the patient's mandible is stabilized with just one of the dentist's hands. Thus, possibly the bimanual technique generates a lower chance of error during the manipulation of the individual's mandible.

Two articles showed no difference between the CR techniques based on the reproducibility of the condylar positions in CR; 8 12 and only one article showed that the chin point guidance technique generated greater reproducibility of the condylar positions in CR than the bimanual technique. 9 Therefore, when evaluating the results of the six articles, 7 8 9 10 11 12 it is possible to suggest that the bimanual technique is more advantageous for the dentist.

A very important fact to be highlighted is that the CR techniques of the guided method are more dependent on the dentist than the CR techniques of the graphical and physiological methods. 2 Thus, the greater the dentist's experience with a RC technique belonging to the guided method, the greater the chance that this type of RC technique is performed correctly.

Only Teo and Wise (supine) and Keshvad and Winstanley (upright) reported the position of patients during CR records. 9 11 Furthermore, only Keshvad and Winstanley and Hobo and Iwata reported that one operator performed all CR techniques in their studies. 10 11 The position of the patient (supine or upright) during CR recording is an important factor, since it may influence his or her mandibular position (i.e., the patient's mandible may be more posterior after performing a CR technique when patient is in the supine position than when he or she is in vertical position, due to the force of gravity). 17 19 20 21 In addition, for a guided method technique, different operators may apply different “levels of force” to an individual's chin, and this can influence the reproducibility results of the mandibular position in CR (i.e., greater chance of mandibular position variability). Therefore, in a study comparing CR techniques, it is essential that only one operator performs all CR techniques, and that all research participants are seated (upright position) or supine during the execution of CR techniques.

Only two articles reported that CR techniques were performed at approximately the same time of day. 11 12 However, these articles do not report whether the records were made in the morning, afternoon, or evening. According to studies in the literature, 22 23 the period of the day (morning, afternoon, or evening) may have an influence on condylar positions after performing a CR technique. 22 23 Therefore, in a clinical study, CR recordings should be performed in a single period of the day, that is, in the morning, afternoon or evening, and at approximately the same times within one of these periods. 2 In addition, this information is important to facilitate the comparison of findings between studies. 2

Only Keshvad and Winstanley and Galeković et al reported the time intervals in which CR recordings were performed ( Table 4 ). 11 12 This information is very relevant as it indicates that at each time point all CR techniques were performed once. Furthermore, as all CR techniques were performed at each time point, they all received the same influence from the patient's emotional and muscle factors at each time point. Thus, this promotes methodological standardization, helping to avoid bias.

To achieve the CR, a deprogramming of proprioceptive memory and some degree of muscle relaxation are needed. Thus, to achieve these goals, it is possible to use the Lucia jig technique for a few minutes in the patient's mouth before performing a CR technique. 24 25 26 27 This device avoids occlusal contacts of the posterior teeth, causing a deprogramming of the proprioceptive memory of the periodontal ligament and promoting the relaxation of the masticatory muscles. 26 These factors facilitate the manipulation of the individual's jaw, helping to avoid its deviation to an incorrect position during the execution of a CR technique. 26 Consequently, this contributes to obtaining a correct CR record. 26 The six articles included in this systematic review reported the use of a jig ( Table 3 ). However, the time of use of this type of device by the participants of each study was only reported by Teo and Wise (10 minutes), Hobo and Iwata (20 minutes), Keshvad and Winstanley (15 minutes), and Galeković et al (5 minutes). 9 10 11 12 The present systematic review recommends that future articles report how many minutes the participants used their jigs, as this allows for a more accurate comparison between articles.

Limitations of this review include the lack of information in the articles included regarding the number of operators, patient position, etc ( Table 4 ); the lack of standardization of the devices used to compare the CR techniques, which prevented a statistical comparison between the articles; the lack of studies comparing graphical or physiological techniques; and the lack of studies evaluating completely edentulous individuals. Therefore, further clinical studies are recommended comparing the CR techniques evaluated in this systematic review, based on the reproducibility of the condylar positions in CR.

The findings and limitations of the present systematic review can help to establish the parameters for future clinical studies comparing CR techniques.

## Conclusion

In this systematic review, the bimanual technique was often superior (generated greater reproducibility of the CR) or at least equivalent to the chin point guidance technique. Therefore, for individuals with complete dentition and without temporomandibular disorders, the bimanual technique is more recommended.
